# Development of a deep learning model using multimodal data for dementia diagnosis

**DOI:** 10.1002/alz70856_099536

**Published:** 2025-12-24

**Authors:** Hee Won Yang, Minwoo Cho, Jeong Lan Kim

**Affiliations:** ^1^ Chungnam National University Hospital, Daejeon, Daejeon, Korea, Republic of (South); ^2^ Department of Transdisciplinary Medicine, Seoul National University Hospital, Seoul, Korea, Republic of (South); ^3^ School of Medicine, Chungnam National University, Daejeon, Korea, Republic of (South)

## Abstract

**Background:**

This study aims to develop a multimodal deep learning model that integrates voice and drawing data collected during dementia screening tests to improve the accuracy of dementia diagnosis. The study also evaluates the impact of different data modalities on classification performance.

**Method:**

1,091 participants (normal cognition, mild cognitive impairment, dementia) were included from five university hospitals located in different regions of South Korea. Voice responses were converted into Mel Frequency Cepstral Coefficient (MFCC) spectrograms, and pentagon drawings were preprocessed into grayscale images. DenseNet was used for feature extraction from voice and drawing data, while demographic and clinical data were analyzed using a multilayer perceptron (MLP). The final multimodal model combined these modalities using weighted ensemble learning.

**Result:**

The multimodal model achieved an accuracy of 66.3% (95% CI: 61.9–70.7) and an AUC of 0.73 (95% CI: 0.70–0.76) for three‐group classification (normal, MCI, dementia). For binary classification (normal vs. dementia), the model achieved an accuracy of 86.9% (95% CI: 84.3–89.4) and an AUC of 0.86 (95% CI: 0.84–0.88). Voice data alone showed strong diagnostic performance, with comparable accuracy and AUC to multimodal models. Drawing data improved performance in multi‐class classification but had limited impact in binary tasks. Clinical data, including MMSE scores and demographic information, provided modest additional contributions to overall model performance.

**Conclusion:**

The multimodal deep learning model combining voice, drawing, and clinical data demonstrated promising performance in diagnosing cognitive impairment and dementia. These findings suggest that integrating diverse data modalities can enhance diagnostic accuracy and provide a scalable approach for early detection in clinical and real‐world settings.

